# Maternal hypertensive disorders and neurodevelopmental disorders in offspring: a population-based cohort in two Nordic countries

**DOI:** 10.1007/s10654-021-00756-2

**Published:** 2021-05-04

**Authors:** Hui Wang, Krisztina D. László, Mika Gissler, Fei Li, Jun Zhang, Yongfu Yu, Jiong Li

**Affiliations:** 1 MOE-Shanghai Key Laboratory of Children’s Environmental Health, Xin Hua Hospital Affiliated to Shanghai Jiao Tong University School of Medicine, Shanghai, China; 2 Department of Clinical Medicine-Department of Clinical Epidemiology, Aarhus University Hospital, Olof Palmes Alle 43-45, 8200 Aarhus N, Denmark; 3 Department of Global Public Health, Karolinska Institutet, Stockholm, Sweden; 4 Information Services Department, Finnish Institute for Health and Welfare, Helsinki, Finland; 5 Research Center for Child Psychiatry, University of Turku, Turku, Finland; 6 Department of Neurobiology, Care Sciences and Society, Karolinska Institute, Stockholm, Sweden; 7 Department of Developmental and Behavioral Pediatric and Child Primary Care, Xin Hua Hospital Affiliated to Shanghai Jiao Tong University School of Medicine, Shanghai, China; 8 Department of Biostatistics, School of Public Health, and the Key Laboratory of Public Health Safety of Ministry of Education, Fudan University, Shanghai, China

**Keywords:** Hypertensive disorders during pregnancy, Pre-eclampsia, Attention-deficit/hyperactivity disorder, Autism spectrum disorder, Intellectual disability, Register-based research

## Abstract

Maternal hypertensive disorders during pregnancy (HDP) have been associated with neuropsychiatric problems in offspring. We aim to investigate the associations between specific types of maternal HDP and offspring neurodevelopmental disorders and further examine whether the timing of onset and severity of HDP would affect these associations. The study population consisted of 4,489,044 live-born singletons in Denmark during 1978–2012 and Sweden during 1987–2010. Maternal HDP was categorized into chronic hypertension, gestational hypertension, and pre-eclampsia; pre-eclampsia was further stratified according to timing (early-onset, late-onset), or severity (moderate, severe) of the disease. Neurodevelopmental disorders, including attention-deficit/hyperactivity disorder (ADHD), autism spectrum disorder (ASD), and intellectual disability (ID), were defined by ICD-coded register diagnosis. Cox regression was used to calculate hazard ratios (HR) while adjusting for potential confounders, and sibling analyses assessed the influence of unmeasured shared familial factors. Maternal HDP was associated with increased risks of ADHD (HR, 1.24; 95% confidence interval [CI], 1.20–1.28), ASD (1.29 [1.24–1.34]), and ID (1.58 [1.50–1.66]) in offspring, respectively, which was mostly driven by pre-eclampsia. The strongest associations were observed for early-onset and severe pre-eclampsia, and the corresponding HRs for ADHD, ASD and ID were 1.93 [1.73–2.16], 1.86 [1.61–2.15], and 3.99 [3.42–4.65], respectively. The results were similar in the sibling analyses. The associations between maternal HDP and offspring neurodevelopmental disorders were consistent across the subgroups of sex, preterm status, parity, maternal age and psychiatric disorders. Maternal HDP, especially early-onset pre-eclampsia, are associated with increased risks of ADHD, ASD, and ID in particular, independent of shared familial factors.

**Supplementary Information:**

The online version contains supplementary material available at 10.1007/s10654-021-00756-2.

## Introduction

Neurodevelopmental disorders, such as attention-deficit/hyperactivity disorder (ADHD) and autism spectrum disorder (ASD), affect about 10% of children worldwide [1]. Neurodevelopmental disorders can lead to a range of adverse outcomes [2–5], including difficulties in academic and career development [3], suicide [4], and premature death [5]. Given the increasing rate of neurodevelopmental disorders [6], gaining knowledge about their etiology is a public health priority. In addition to the genetic susceptibility [7], emerging evidence has indicated that exposure to environmental factors [8], especially in utero [9], may contribute to their development.

Hypertensive disorders during pregnancy (HDP) complicate 5–10% of all pregnancies [10]. HDP may affect not only maternal health and pregnancy outcomes [11], but also predispose the offspring to neurobehavioral disorders later in life through impacting developing fetal brain due to poor vascularization of the placenta [12, 13]. Previous studies have reported that maternal HDP is associated with increased risks of ADHD [14], ASD [15, 16], cerebral palsy [17], and emotional and behavioral problems in offspring [18–21]. However, HDP is a group of conditions that include chronic hypertension, gestational hypertension, and pre-eclampsia/eclampsia (de novo or superimposed on chronic hypertension) [22]. Studies so far have mainly focused on pre-eclampsia [16, 17, 19, 21], evidence on other types of HDP is lacking [15, 20]. One recent population-based study found that maternal HDP was associated with increased risks of mental disorders in offspring, but the timing of pre-eclampsia and unmeasured familiar (genetic or environmental) risk factors were not taken into consideration [23]. As such, there is a need to evaluate whether unmeasured familial factors exert an influence on the associations between maternal HDP and neurodevelopmental disorders in offspring.

Taking advantage of national Danish and Swedish registers, we aimed to investigate the associations between specific types of maternal HDP and offspring neurodevelopmental disorders using population-based cohort and sibling designs, accounting for unmeasured familial confounding. We further examined whether the timing of onset and severity of HDP would affect these associations [13, 16, 17].

## Methods

### Design and population

We conducted a nationwide cohort study using data from several registers in Denmark [24–30] and Sweden [31–34]. The description of the registers is provided in Table S1. In both countries, all live births have a unique personal identification number that permits an accurate linkage of individual-level data. We identified all singleton live births in Denmark from 1978 to 2012 (n = 2,098,944) and in Sweden from 1987 to 2010 (n = 2,402,666). After excluding children who had extreme gestational age (< 154 or > 315 days) (n = 461 in Denmark and n = 122 in Sweden), without information on sex (n = 86 in Denmark and n = 14 in Sweden), and/or with chromosomal abnormalities (n = 7782 in Denmark and n = 4101 in Sweden), the final analysis included 2,090,615 children in Denmark and 2,398,429 children in Sweden. We followed them from birth until the date of the first diagnosis of a specific neurodevelopmental disorder, emigration, death, 18-year birthday, or end of follow-up (December 31, 2016 in Denmark and December 31, 2014 in Sweden), whichever came first.

In both Denmark and Sweden, prenatal care is standardized and free of charge, which includes visits every fourth week up to 24 gestational weeks, then every second week to 36 weeks, and weekly thereafter. Information on the maternal medical and obstetric history is collected with an interview during the first antenatal visit (around 12 gestational weeks) [30, 33]. After delivery, the responsible doctor would record each women’s diseases and complications during pregnancy and delivery, according to the appropriate *ICD* codes. The Medical Birth Registries (MBR) in both countries contain detailed information on maternal disease history and offspring birth outcomes. As in other studies [35–37], we have used MBR supplemented by National Patient Register (NPR) to extract information on maternal HDP, which has been considered highly reliable [29, 33].

The health care system in both countries is government-funded and ensures equal and free of charge access to hospital care [28, 30, 31, 34]. The doctors register all diagnoses associated with each hospital contact, and the reporting is mandatory by Danish and Swedish legislation, ensuring nationwide and almost complete coverage. We have used NPR supplemented by Psychiatric Central Research Registry to extract data on the diagnosis of maternal HDP (chronic hypertension, pre-eclampsia, gestational hypertension) and other disorders, as well as child neurodevelopmental disorders [5, 38].

### Assessment of exposures

Information on maternal HDP was obtained from the combination of the Medical Birth Register (MBR) and the Danish National Patient Register (DNPR) in Denmark [28, 29], and the MBR and the Swedish National Patient Register (SNPR) in Sweden [33, 34]. Maternal HDP was categorized into three subtypes of chronic hypertension, gestational hypertension, and pre-eclampsia. We grouped eclampsia and the HELLP syndrome with pre-eclampsia because these conditions are rare as shown in previous studies [35, 36]. If women had more than one diagnosis of hypertensive disorders, we categorized them according to the following hierarchy: chronic hypertension, pre-eclampsia, and gestational hypertension [37].

Pre-eclampsia was divided into early-onset and late-onset pre-eclampsia according to the current consensus of disease definition of early-onset pre-eclampsia (early-onset pre-eclampsia: < 34 completed gestational weeks; late-onset pre-eclampsia: ≥ 34 completed gestational weeks) [39]. In Sweden, information on the timing of onset of pre-eclampsia is not available, pre-eclampsia was considered as early-onset when delivery occurred before 34 completed gestational weeks [36]. Pre-eclampsia was further categorized into severe pre-eclampsia (including eclampsia and the HELLP syndrome) and moderate pre-eclampsia [35–37]. Detailed description of the method used to identify HDP is provided in Table S2.

### Ascertainment of outcomes

Information on diagnosis of ADHD, ASD and intellectual disability (ID) was obtained from the DNPR and the Danish Psychiatric Central Research Register in Denmark [25, 28], and from the SNPR in Sweden [34]. The *International Statistical Classification of Diseases and Related Health Problems (ICD)* codes are available in Table S3 [38, 40, 41]. When investigating the specific neurodevelopmental disorders, we defined the date of onset as the first day of each specific diagnosis, irrespective of other neurodevelopmental disorders diagnoses, if existed.

### Covariates

Based on previous research [13, 16, 18, 21], the following variables were considered as potential confounders: sex of the child (male, female), calendar period of birth (a 5-year interval during 1978–2012 in Denmark and a 4-year interval during 1987–2010 in Sweden), parity (1, 2, ≥ 3), maternal age at birth (≤ 25, 26–30, 31–35, ≥ 36 years), maternal country of origin (Denmark/Sweden, other countries), maternal education level (0–9, 10–14, ≥ 15 years), maternal cohabitation status at birth (yes, no), and maternal psychiatric disorder before the childbirth (yes, no).

### Statistical analysis

Cox proportional hazards regression model with the child’s age as the time scale was used to estimate the hazard ratio (HR) with 95% confidence intervals (CI) for the association of maternal HDP with the risk of offspring neurodevelopmental disorders, taking the timing of onset and severity of HDP into account. In the final model, adjustments were made for sex, calendar year of birth, parity, maternal age at birth, maternal education level, maternal cohabitation, and maternal psychiatric disorders before the childbirth. We used the robust sandwich estimator for standard errors to account for the clustering of individuals within nuclear families bound by the same biological mother. Kaplan–Meier curves were used to illustrate the probability of the neurodevelopmental disorders diagnoses in exposed and unexposed groups.

To account for unmeasured familial confounding, we performed sibling comparison analysis in which only sibling pairs discordant for both maternal HDP and offspring neurodevelopmental disorders contributed to the effect estimate [42]. As chronic hypertension would not change between pregnancies and the number of gestational hypertension is small, the sibling comparison model was appropriate for pre-eclampsia that could vary among siblings born to the same mother in the analysis.

We performed several sensitivity analyses. First, we tested whether the associations varied by the sex of the child, parity, preterm birth, and maternal factors, including maternal age and psychiatric disorders. Second, in order to assess potential effects of neonatal complications, we performed the analyses after excluding children with preterm birth (< 37 gestational weeks), low birth weight (< 2500 g), and low Apgar score at 5 min (< 7) [43]. Third, we performed mediation analyses to examine whether gestational age mediated the observed association by calculating direct and indirect effects in the STATA modulate PARAMED. Fourth, given the change in *ICD* revisions (*ICD*-10 was adopted in 1994 in Denmark and in 1997 in Sweden) [28, 31] and the offspring neurodevelopmental disorders identification strategy (all outpatient diagnosis were available since 1995 in Denmark and all specialized outpatient care were available since 2001 in Sweden) [25, 31], we restricted the analysis to offspring born after 1995 in Denmark and 2001 in Sweden. Fifth, we applied multiple imputation procedure by chained equations to impute ten replications to handle missing values on covariates (i.e., maternal education level and cohabitation status). Sixth, to address concerns about the validity of early neurodevelopmental diagnoses, we performed analysis in subsamples excluding offspring with diagnoses before the age of 3 years. Seventh, we additionally included superimposed pre-eclampsia as a separate group of exposure. Eighth, we also used the Swedish approach (pre-eclampsia was considered as early-onset when delivery occurred before 34 completed gestational weeks) of defining early-onset pre-eclampsia in the Danish participants to check whether the approaches for the definition of early-onset pre-eclampsia in the two countries is comparable. Ninth, we performed analyses excluding individuals with chronic hypertension to check whether the overall estimation of the association between maternal HDP and offspring outcomes will be affected. Tenth, we further categorized chronic hypertension into two subgroups: chronic hypertension from primary diagnosis or chronic hypertension from secondary diagnosis. Eleventh, we additionally adjusted the Charlson Comorbidity Index (CCI) scores (0 [low] referring to no recorded underlying diseases implemented in the CCI; 1–2 [moderate]; and > 2 [high]) [44–47]. Lastly, to assess whether maternal pre-pregnancy body mass index (BMI) confounded the associations, the analyses were restricted to offspring born after 2004 when maternal BMI became available. Maternal pre-pregnancy BMI was classified into underweight (< 18.5 kg/m^2^), normal weight (18.5–24.9 kg/m^2^), overweight (25.0–29.9 kg/m^2^), and obese (≥ 30 kg/m^2^). All statistical analyses were performed using Stata, version 15.1 (StataCorp).

## Results

### Descriptive statistics stratified by maternal HDP

During the study period, we identified 184 033 (4.1%) individuals born to mothers with HDP (chronic hypertension: 0.6%, gestational hypertension: 0.8%, and pre-eclampsia: 2.7%). In Denmark, 89 240 (4.3%) were born to mothers with HDP (chronic hypertension: 0.7%, gestational hypertension: 0.8% and pre-eclampsia: 2.8%) and in Sweden, 94 793 (4.0%) were born to mothers with HDP (chronic hypertension: 0.5%, gestational hypertension: 0.9% and pre-eclampsia: 2.6%). Compared with unexposed offspring, exposed offspring were more likely to be born preterm or had lower Apgar scores at 5 min. Mothers of exposed offspring were more likely to be older, to have a higher parity, or more comorbid psychiatric disorders (Table 1).

**Table 1:** Characteristics of the study population at baseline according to maternal hypertensive disorders during pregnancy

	Denmark		Sweden	
	No	Yes	No	Yes
Sex				
Boys	1,026,939 (51.3)	46,386 (51.9)	1,183,231 (51.4)	49,580 (52.3)
Girls	974,436 (48.7)	42,854 (48.1)	1,120,405 (48.6)	45,213 (47.7)
Preterm birth (< 37 gestational weeks)				
No	1,839,865 (91.9)	77,381 (86.7)	2,199,179 (95.5)	77,495 (81.8)
Yes	84,043 (4.2)	11,684 (13.1)	101,826 (4.4)	17,173 (18.1)
Missing	77,467 (3.9)	175 (0.2)	2631 (0.1)	128 (0.1)
Apgar score at 5 min				
10	1,836,432 (91.8)	78,005 (87.4)	1,913,306 (83.0)	70,164 (74.0)
≤ 9	132,835 (6.6)	10,387 (11.6)	368,200 (16.0)	23,603 (24.9)
Missing	32,108 (1.6)	848 (1.0)	22,130 (1.0)	1026 (1.1)
Parity				
1	878,604 (43.9)	52,237 (58.5)	969,936 (42.1)	56,816 (59.9)
2	753,614 (37.6)	24,265 (27.2)	841,565 (36.5)	23,021 (24.3)
≥ 3	369,157 (18.5)	12,738 (14.3)	492,135 (21.4)	14,956 (15.8)
Maternal age (years)				
≤ 24	421,987 (21.0)	19,248 (21.6)	441,908 (19.2)	18,262 (19.3)
25–29	742,193 (37.1)	31,224 (35.0)	785,823 (34.1)	29,802 (31.4)
30–34	587,804 (29.4)	24,475 (27.4)	711,839 (30.9)	27,693 (29.2)
≥ 35	249,391 (12.5)	14,293 (16.0)	364,066 (15.8)	19,036 (20.1)
Maternal education (years)				
≤ 9	558,202 (27.9)	25,158 (28.2)	799,080 (34.7)	33,214 (35.0)
10–15	873,455 (43.6)	40,410 (45.3)	1,121,677 (48.7)	48,668 (51.3)
≥ 15	557,226 (26.9)	22,744 (25.5)	324,747 (14.1)	11,440 (12.1)
Missing	32,492 (1.6)	928 (1.0)	57,132 (2.5)	1471 (1.6)
Maternal psychiatric disorders				
No	1,897,588 (94.8)	84,006 (94.1)	2,219,248 (96.3)	90,934 (95.9)
Yes	103,787 (5.2)	5234 (5.9)	84,388 (3.7)	3859 (4.1)
Maternal cohabitation at birth				
Yes	1,123,132 (56.1)	46,211 (51.8)	1,232,307 (53.5)	56,301 (59.4)
No	876,533 (43.8)	43,004 (48.2)	1,062,179 (46.1)	38,291 (40.4)
Missing	1710 (0.1)	25 (< 0.1)	9150 (0.4)	201 (0.2)
Attention-deficit/hyperactivity disorders				
Yes	31,629 (1.6)	1629 (1.8)	58,252 (2.5)	2966 (3.1)
No	1,969,746 (98.4)	87,611 (98.2)	2,245,384 (97.5)	91,827 (96.9)
Autism spectrum disorders				
Yes	22,536 (1.1)	1306 (1.5)	27,654 (1.2)	1571 (1.7)
No	1,978,838 (98.9)	87,934 (98.5)	2,275,982 (98.8)	93,222 (98.3)
Intellectual disability				
Yes	10,305 (0.5)	620 (0.7)	16,364 (0.7)	1042 (1.1)
No	1,991,040 (99.5)	88,620 (99.3)	2,287,272 (99.3)	93,751 (98.9)

**Fig. 1:**
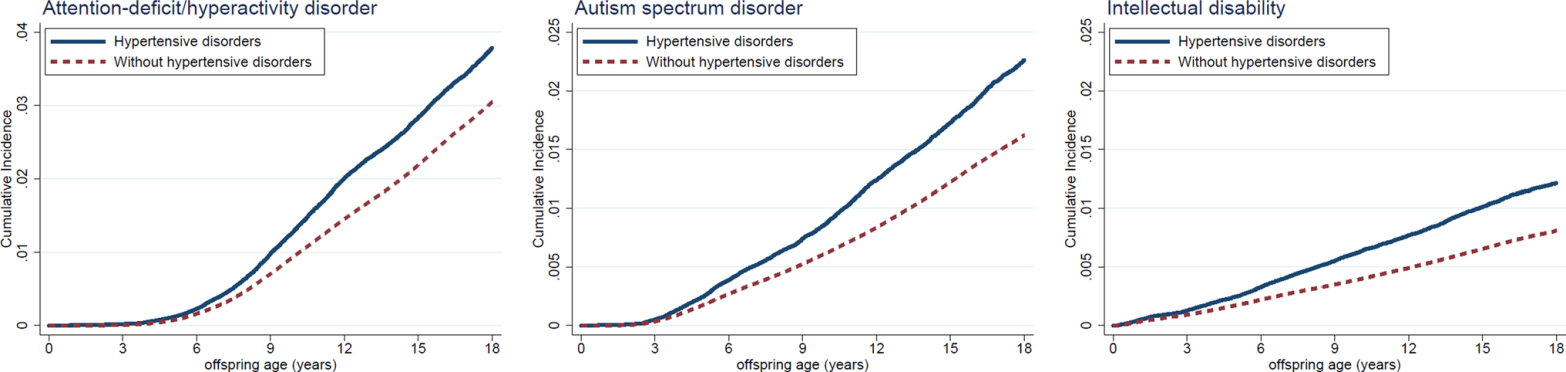
Cumulative risk for neurodevelopmental disorders in children after exposure to maternal hypotensive disorders during pregnancy

Compared with unexposed offspring, exposed offspring had a higher risk of developing neurodevelopmental disorders (Fig. 1). Among offspring exposed to maternal HDP, the estimates of cumulative incidences by 18 years of age were 3.78% (95% CI 3.67–3.89%) for ADHD, 2.26% (95% CI 2.18–2.35%) for ASD, and 1.21% (95% CI 1.15–1.27%) for ID, which are higher than those among unexposed offspring (3.05% [95% CI 3.03–3.07%] for ADHD, 1.62% [95% CI 1.61–1.63%] for ASD, and 0.81% [95% CI 0.80–0.82%] for ID, respectively). The cumulative incidences for the neurodevelopmental disorders by countries showed similar patterns (Figure S1–2).

### Population-wide association and comparison of timing and severity of maternal HDP

At the whole population level, maternal HDP was associated with increased risks of ADHD (HR, 1.24; 95% CI 1.20–1.28), ASD (HR, 1.29; 95% CI 1.24–1.34), and ID (HR, 1.58; 95% CI 1.50–1.66). When exploring the risk by types of HDP, we found the overall estimation was mostly driven by maternal pre-eclampsia. The HRs in offspring exposed to maternal pre-eclampsia were 1.27 (95% CI 1.23–1.32) for ADHD, 1.36 (95% CI 1.30–1.42) for ASD, and 1.70 (95% CI 1.60–1.81) for ID, respectively (Tables 2, 3, 4).

**Table 2:** Associations between maternal hypertensive disorders and attention-deficit/hyperactivity disorders in offspring

	Attention-deficit/hyperactivity disorders					
	Combined		Denmark		Sweden	
	Cases (No.)	aHR^a^ (95% CI)	Cases (No.)	aHR^a^ (95% CI)	Cases (No.)	aHR^a^ (95% CI)
Maternal hypertensive disorders						
No hypertensive disorders	89,881	1.00 (ref)	31,629	1.00 (ref)	58,252	1.00 (ref)
Hypertensive disorders	4595	1.24 (1.20–1.28)	1629	1.16 (1.10–1.22)	2966	1.23 (1.18–1.27)
Chronic hypertension	584	1.23 (1.13–1.34)	261	1.04 (0.91–1.17)	323	1.36 (1.22–1.52)
Gestational hypertension	829	1.13 (1.05–1.21)	259	1.06 (0.94–1.20)	570	1.14 (1.05–1.24)
Pre-eclampsia	3182	1.27 (1.23–1.32)	1109	1.22 (1.15–1.30)	2073	1.23 (1.18–1.29)
By timing of pre-eclampsia						
Early-onset	506	1.77 (1.62–1.94)	230	1.51 (1.32–1.72)	276	1.94 (1.71–2.19)
Late-onset	2676	1.21 (1.16–1.26)	879	1.17 (1.09–1.25)	1797	1.16 (1.11–1.22)
By severity of pre-eclampsia						
Moderate	2223	1.22 (1.17–1.27)	822	1.15 (1.07–1.24)	1401	1.18 (1.12–1.25)
Severe	959	1.41 (1.32–1.51)	287	1.48 (1.31–1.66)	672	1.35 (1.24–1.46)
By timing # severity						
Early-onset # Severe pre-eclampsia	329	1.93 (1.73–2.16)	114	1.84 (1.53–2.22)	215	1.88 (1.64–2.16)
Early-onset # Moderate pre-eclampsia	177	1.54 (1.32–1.79)	116	1.28 (1.06–1.54)	61	2.16 (1.67–2.80)
Late-onset # Severe pre-eclampsia	630	1.24 (1.14–1.34)	173	1.30 (1.12–1.52)	457	1.19 (1.08–1.30)
Late-onset # Moderate pre-eclampsia	2046	1.20 (1.15–1.25)	706	1.13 (1.05–1.22)	1340	1.16 (1.10–1.22)

*aHR* adjusted hazard ratio, *CI* confidence interval, *No.* number

^a^Adjusted for sex, calendar year, parity, maternal age, maternal education, maternal cohabitation at birth, maternal history of psychiatric disorders before childbirth

**Table 3:** Associations between maternal hypertensive disorders and autism spectrum disorders in offspring

	Autism spectrum disorders					
	Combined		Denmark		Sweden	
	Cases (No.)	aHR^a^ (95% CI)	Cases (No.)	aHR^a^ (95% CI)	Cases (No.)	aHR^a^ (95% CI)
Maternal hypertensive disorders						
No hypertensive disorders	50,190	1.00 (ref)	22,536	1.00 (ref)	27,654	1.00 (ref)
Hypertensive disorders	2877	1.29 (1.24–1.34)	1306	1.23 (1.16–1.30)	1571	1.30 (1.24–1.37)
Chronic hypertension	388	1.18 (1.07–1.31)	236	1.19 (1.05–1.36)	152	1.12 (0.95–1.32)
Gestational hypertension	508	1.15 (1.05–1.26)	207	1.07 (0.93–1.23)	301	1.20 (1.06–1.35)
Pre-eclampsia	1981	1.36 (1.30–1.42)	863	1.29 (1.20–1.38)	1118	1.36 (1.28–1.45)
By timing of pre-eclampsia						
Early-onset	313	1.74 (1.55–1.95)	163	1.42 (1.21–1.66)	150	2.13 (1.81–2.52)
Late-onset	1668	1.30 (1.24–1.37)	700	1.26 (1.17–1.36)	968	1.29 (1.21–1.38)
By severity of pre-eclampsia						
Moderate	1413	1.33 (1.26–1.40)	665	1.28 (1.18–1.39)	748	1.30 (1.21–1.41)
Severe	568	1.44 (1.33–1.57)	198	1.31 (1.14–1.51)	370	1.50 (1.35–1.67)
By timing # severity						
Early-onset # severe pre-eclampsia	189	1.86 (1.61–2.15)	68	1.44 (1.13–1.83)	121	2.16 (1.79–2.59)
Early-onset # moderate pre-eclampsia	124	1.58 (1.33–1.89)	95	1.40 (1.14–1.72)	29	2.05 (1.40–2.99)
Late-onset # severe pre-eclampsia	379	1.30 (1.17–1.44)	130	1.25 (1.05–1.49)	249	1.31 (1.15–1.49)
Late-onset # moderate pre-eclampsia	1289	1.31 (1.23–1.38)	570	1.26 (1.16–1.38)	719	1.29 (1.19–1.39)

*aHR* adjusted hazard ratio, *CI* confidence interval, *No.* number

^a^Adjusted for sex, calendar year, parity, maternal age, maternal education, maternal cohabitation at birth, maternal history of psychiatric disorders before childbirth

**Table 4:** Associations between maternal hypertensive disorders and intellectual disability in offspring

	Intellectual disability					
	Combined		Denmark		Sweden	
	Cases (No.)	aHR^a^ (95% CI)	Cases (No.)	aHR^a^ (95% CI)	Cases (No.)	aHR^a^ (95% CI)
Maternal hypertensive disorders						
No hypertensive disorders	26,669	1.00 (ref)	10,305	1.00 (ref)	16,364	1.00 (ref)
Hypertensive disorders	1662	1.58 (1.50–1.66)	620	1.41 (1.30–1.54)	1042	1.64 (1.53–1.75)
Chronic hypertension	178	1.41 (1.21–1.64)	73	1.18 (0.93–1.49)	105	1.60 (1.31–1.96)
Gestational hypertension	285	1.28 (1.13–1.45)	111	1.32 (1.08–1.60)	174	1.21 (1.03–1.43)
Pre-eclampsia	1199	1.70 (1.60–1.81)	436	1.49 (1.35–1.64)	763	1.77 (1.64–1.92)
By timing of pre-eclampsia						
Early-onset	284	3.69 (3.27–4.17)	116	2.74 (2.26–3.30)	168	4.54 (3.86–5.33)
Late-onset	915	1.46 (1.36–1.56)	320	1.27 (1.13–1.43)	595	1.51 (1.39–1.65)
By severity of pre-eclampsia						
Moderate	796	1.51 (1.40–1.63)	308	1.30 (1.16–1.47)	488	1.60 (1.46–1.76)
Severe	403	2.27 (2.05–2.52)	128	2.25 (1.88–2.70)	275	2.19 (1.93–2.49)
By timing # severity						
Early-onset # severe pre-eclampsia	183	3.99 (3.42–4.65)	68	3.89 (3.04–4.99)	115	3.74 (3.06–4.56)
Early-onset # moderate pre-eclampsia	101	3.28 (2.69–4.00)	48	1.95 (1.46–2.60)	53	7.72 (5.86–10.16)
Late-onset # severe pre-eclampsia	220	1.69 (1.47–1.94)	60	1.53 (1.18–1.99)	160	1.71 (1.45–2.02)
Late-onset # moderate pre-eclampsia	695	1.39 (1.29–1.51)	260	1.23 (1.08–1.39)	435	1.45 (1.31–1.60)

*aHR* adjusted hazard ratio, *CI* confidence interval, *No.* number

^a^Adjusted for sex, calendar year, parity, maternal age, maternal education, maternal cohabitation at birth, maternal history of psychiatric disorders before childbirth

Children born to mothers with early-onset pre-eclampsia had particularly high risks of ADHD (HR, 1.77; 95% CI 1.62–1.94), ASD (HR, 1.74; 95% CI 1.55–1.95), and ID (HR, 3.69; 95% CI 3.27–4.17). We also observed increased risks among offspring exposed to severe pre-eclampsia, the risk estimates were 1.41 (95% CI 1.32–1.51) for ADHD, 1.44 (95% CI 1.33–1.57) for ASD, and 2.27 (95% CI 2.05–2.52) for ID. It is worth noting that 57.9% of early-onset pre-eclampsia was also diagnosed with severe pre-eclampsia. Therefore, we further examined the associations according to both timing of onset and severity of pre-eclampsia. The association between early-onset pre-eclampsia and the outcomes was slightly affected by severity of pre-eclampsia. For ADHD, the HR was 1.93 (95% CI 1.73–2.16) for early-onset and severe pre-eclampsia, and 1.54 (95% CI 1.32–1.79) for early-onset and moderate pre-eclampsia. On the other hand, the association between late-onset pre-eclampsia and the outcomes was similar across the severity of pre-eclampsia. For ADHD, the HR was 1.24 (95% CI 1.14–1.34) for late-onset and severe pre-eclampsia, and 1.20 (95% CI 1.15–1.25) for late-onset and moderate pre-eclampsia. The same pattern of results was found for ASD and ID (Tables 2, 3, 4)).

### Sibling comparisons

For the sibling comparison analyses, we identified a subsample of 1,481,847 families with at least 2 siblings. Of these families, 12,490 (0.84%) included sibling discordant for pre-eclampsia and ADHD, 6804 (0.46%) included sibling discordant for pre-eclampsia and ASD, and 4313 (0.29%) included sibling discordant for pre-eclampsia and ID. In the sibling comparison models, maternal pre-eclampsia was associated with ADHD (HR, 1.19; 95% CI 1.11–1.28), ASD (HR, 1.36; 95% CI 1.24–1.49), and ID (HR, 1.43; 95% CI 1.27–1.61) (Table 5).

**Table 5:** Associations between maternal pre-eclampsia and neurodevelopmental disorders in offspring using sibling analyses

Neurodevelopmental disorders	Combined		Denmark		Sweden	
	No. of cases	Adjusted^a^ HR (95% CI)	No. of cases	Adjusted^a^ HR (95% CI)	No. of cases	Adjusted^a^ HR (95% CI)
*For ADHD*						
No pre-eclampsia	2170	1.00 (ref)	851	1.00 (ref)	1319	1.00 (ref)
Maternal pre-eclampsia	1746	1.19 (1.11–1.28)	633	1.16 (1.02–1.33)	1113	1.20 (1.08–1.33)
By timing of onset						
Early-onset	233	1.54 (1.34–1.77)	129	1.40 (1.14–1.72)	104	1.80 (1.44–2.26)
Late-onset	1513	1.15 (1.07–1.24)	504	1.12 (0.97–1.28)	1009	1.16 (1.05–1.29)
By severity						
Moderate	1270	1.16 (1.08–1.25)	483	1.12 (0.98–1.29)	787	1.18 (1.06–1.31)
Severe	476	1.29 (1.16–1.43)	150	1.32 (1.09–1.60)	326	1.26 (1.09–1.46)
*For ASD*						
No pre-eclampsia	1172		540		632	1.00 (ref)
Maternal pre-eclampsia	1076	1.36 (1.24–1.49)	494	1.40 (1.19–1.65)	582	1.29 (1.11–1.49)
By timing of onset						
Early-onset	134	1.61 (1.34–1.94)	77	1.41 (1.19–1.66)	57	2.27 (1.68–3.06)
Late-onset	942	1.33 (1.21–1.46)	417	1.38 (1.05–1.80)	525	1.23 (1.06–1.43)
By severity						
Moderate	809	1.32 (1.20–1.46)	395	1.37 (1.16–1.62)	414	1.25 (1.07–1.46)
Severe	267	1.49 (1.30–1.72)	99	1.52 (1.20–1.93)	168	1.41 (1.15–1.73)
*For ID*						
No pre-eclampsia	720	1.00 (ref)	322	1.00 (ref)	398	1.00 (ref)
Maternal pre-eclampsia	653	1.43 (1.27–1.61)	254	1.29 (1.04–1.60)	399	1.60 (1.33–1.93)
By timing of onset						
Early-onset	150	2.50 (2.07–3.02)	65	2.13 (1.60–2.85)	85	3.26 (2.47–4.31)
Late-onset	503	1.27 (1.12–1.44)	189	1.13 (0.89–1.43)	314	1.42 (1.17–1.72)
By severity						
Moderate	439	1.31 (1.15–1.49)	185	1.20 (0.95–1.51)	254	1.45 (1.19–1.78)
Severe	214	1.80 (1.52–2.12)	69	1.59 (1.18–2.15)	145	1.98 (1.57–2.51)

*HR* hazard ratio, *CI* confidence interval, *ADHD* attention-deficit/hyperactivity disorder, *ASD* autism spectrum disorder, *ID* intellectual disability

^a^Adjusted for sex, calendar year parity, maternal age, maternal education, maternal cohabitation at birth, maternal history of psychiatric disorders before childbirth

### Sensitivity analyses

Stratification by sex of the offspring, parity, Apgar score at 5 min, maternal age and maternal psychiatric disorders did not indicate any significant differences in the studied associations (Tables S4–8). Analyses excluding offspring with adverse birth outcomes such as preterm birth, low birth weight and low Apgar score yielded the similar estimates (Table S9). The proportions of the total effect mediated by gestational age for the associations between maternal HDP and ADHD, ASD, and ID, were 30.7%, 19.2% and 34.9%, respectively (Table S10). Children exposed to superimposed pre-eclampsia had higher risks of ADHD (HR, 1.56; 95% 1.33–1.82), ASD (HR, 1.48; 95% CI 1.21–1.80), and ID (HR, 1.85; 95% CI 1.38–2.48) (Table S11). When applying the Swedish approach of defining early-onset pre-eclampsia in the Danish participants, the estimates for early-onset pre-eclampsia were similar to those in the Swedish cohort (Table S12). Similar associations were observed in the analyses (1) restricted to offspring born after 1995 in Denmark and 2001 in Sweden (Table S13) when specialized outpatient diagnoses were added, or (2) excluding neurodevelopmental diagnosis before the age of three (Table S14), or (3) using the multiple imputation method (Table S15), or (4) additionally adjusting for maternal pre-pregnancy BMI (Table S16), or (5) excluding individuals with chronic hypertension (Table S17), or (6) categorizing chronic hypertension into two subgroups: primary diagnosis or secondary diagnosis (Table S18), or (7) additionally adjusting for CCI scores (Table S19).

## Discussion

In this large population-based cohort study, we found that all the three types of maternal HDP, including chronic hypertension, gestational hypertension and pre-eclampsia, were associated with 1.2- to 1.6-fold risks of neurodevelopmental disorders in offspring, while the overall associations were mostly driven by pre-eclampsia. The strength of associations varied significantly according to the timing of onset or severity of pre-eclampsia and our findings further indicated that the timing of onset of pre-eclampsia was probably more important than the severity. The consistent results from sibling comparison analysis indicated that the observed associations are unlikely confounded by the shared genetic and environmental factors. Moreover, these results remained unchanged when taking into account of a number of important factors, such as maternal socioeconomic status, birth order, maternal history of psychiatric disorders, and adverse birth outcomes.

### Comparison with other studies and interpretation of results

To our knowledge, this is the largest population-based and sibling comparison study to date to investigate the link between maternal HPD and the risk of neurodevelopmental disorders in offspring. Our findings of increased risk of neurodevelopmental disorders related to maternal HDP are inconsistent with several previous studies which reported null or even a protective association between maternal pre-eclampsia and neurodevelopmental disorders in offspring [20, 48, 49]. For example, a case–control study in Australia, including 465 cases matched with 1313 controls, found no association between pre-eclampsia and ASD [20]; the study used un-validated data on maternal pre-eclampsia. A Japanese study found that maternal pre-eclampsia reduced the risk of autistic disorder in offspring admitted to the neonatal intensive care unit, which is subject to selection bias [48].

Our findings are in line with those in several other previous observational studies, demonstrating increased risks of neurodevelopmental disorders in offspring born to mothers with pre-eclampsia [13, 16–19, 50]. A systematic review showed a 1.2-fold risk of ADHD and a 1.3-fold risk of ASD in offspring of mothers with pre-eclampsia [50]. In a registry study in the US, maternal pre-eclampsia was associated with a 1.8-fold increased risk of ASD in offspring, without adjusting for maternal psychiatric disorders [16]. Another registry study in Finland suggested that maternal pre-eclampsia was associated with 20%, 30% and 50% higher risks of ADHD, ASD and ID, respectively [17]. However, this study was only conducted in term births which may lead to collider bias. The Childhood Autism Risks from Genetics and Environmental cohort in US found that offspring exposed to pre-eclampsia had a 2.3-fold risk of ASD and a 1.4-fold risk for developmental delay [13]. Consistent with our results, the risks increased with greater pre-eclampsia severity, but the number of severe pre-eclampsia was very low (n = 11) [13].

We are only aware of one recent birth cohort study (n = 4743) reporting that maternal severe pre-eclampsia and mild pre-eclampsia were associated with 2.0-fold and 1.4-fold risk of overall mental disorders in offspring, respectively [23]. However, data on timing of pre-eclampsia and specific neurodevelopmental disorders were not reported [23]. It is important to note that early-onset pre-eclampsia was associated with almost twofold to fourfold risk of neurodevelopmental disorders in offspring, and these associations were less affected by severity of pre-eclampsia. This suggests that the timing of onset may play a more important role in the pathophysiology than the severity of the disease [12, 51, 52]. Moreover, our study further supports the previous findings by showing that the observed associations were not modified by maternal socioeconomic status, parity, and maternal history of psychiatric disorders.

Most previous studies have not distinguished between gestational hypertension and pre-eclampsia [14, 48, 53, 54]. One birth cohort study found no association between gestational hypertension (n = 263) and mental disorders in children, with a relatively small number of gestational hypertension cases [23]. Similar to our study, other prospective birth cohort studies found that gestational hypertension with or without pre-eclampsia was associated with increased risks of emotional and behavioral problems [55], and impaired cognitive development [56, 57].

There is currently limited evidence on the association between chronic hypertension and neurodevelopmental disorders in offspring. A registry study in Norway showed that there was no association between maternal chronic hypertension and offspring ADHD [58]. However, the prevalence of chronic hypertension in this Norwegian study was only 0.3%, which is substantially lower than that observed in other Nordic countries [59, 60]. As mentioned by the authors, the diagnostic validity of chronic hypertension has not been formally validated in the Medical Birth Registry of Norway. Thus, the potentially under-diagnosed maternal chronic hypertension would most likely attenuate the associations. However, our findings suggest that maternal chronic hypertension may play a role in the development of neurodevelopmental disorders, especially for intellectual disability. Nevertheless, our finding that maternal chronic hypertension was associated with increased risks of neurodevelopmental disorders needs further investigation, in particular given the fact that there existed great challenges in identifying all cases with chronic hypertension because many mild cases might be managed only in primary care and would not be registered in the NPR or MBR [61, 62].

The underlying mechanism linking maternal HDP and neurodevelopmental disorders remains to be elucidated. The fetal programming theory hypothesizes that adverse fetal environment or insult can affect brain development, which leads to a range of neurodevelopmental disorders [8]. It is possible that hypertension during pregnancy may involve a decrease in oxygen and nutrient supply to the fetus as a result of utero-placental underperfusion and hypoxia [12, 51]. Depleted oxygen and nutrition supply to the fetus may impair fetal brain development and thus contribute to greater risk of emotional and behavioral disorders later in life [63]. In addition, early-onset pre-eclampsia is a disorder of defect placentation, whereas other types of HDP are more a metabolic disorder related to maternal risk factors [22]. Early-onset pre-eclampsia is associated with severe placental insufficiency, which contribute to increased susceptibility to neurodevelopmental disorders in offspring [8]. Previous studies have suggested that the association between pre-eclampsia and mental disorders in children might partly be mediated by preterm birth and fetal growth restriction[23, 43]. With the large sample size and long follow-up, our study had sufficient power to examine the mediation effects on the associations of maternal HDP with specific mental disorders. For the first time, our study confirmed that a substantial portion of the association with ADHD, ASD, and ID was mediated via shortened gestational age.

### Strengths and Limitations

Our study has several strengths. First, the large sample up to four million mother–child pairs made it possible to examine all forms of HDP, and to stratify pre-eclampsia by timing of onset or severity, which are not commonly ascertained in other studies [13, 14, 17]. Second, information on HDP and neurodevelopmental disorders were virtually prospectively collected, which can reduce the possibility of recall bias [28, 32]. Third, the diagnoses recorded for HDP and neurodevelopmental disorders have been demonstrated to have high validity [27, 32, 38]. Fourth, the conclusions were based on population-based and sibling comparison studies that are less susceptible to both measured and unmeasured familial confounding factors.

Some limitations need to be noted. First, as the data from the primary care was not available, it is likely that some mild cases could not be identified through the registers, for example, chronic hypertension is often managed in primary care particularly [62]. However, this misclassification of mild cases as healthy controls would probably attenuate the association [64]. In addition, the estimates from our supplementary analyses, in which only hospitalized cases were included or additional adjustment for comorbid diseases was performed, were similar to those from primary analyses. Second, neurodevelopmental disorders were ascertained from NPR diagnoses, while specialized outpatient data which contain substantial proportion of neurodevelopmental disorders records were only available since 1995 in Denmark and since 2001 in Sweden [31, 38]. Thus, we might have missed the diagnoses for children who were born earlier in our cohort. Nevertheless, analyses restricted to years when both outpatient and inpatient registers were in use did not essentially change our estimates. In addition, a number of studies have been conducted to examine the validity of register-based diagnoses of neurodevelopmental disorders in both Denmark and Sweden, all showing high quality [25, 65–67]. Third, even though sibling analysis was used to address unmeasured stable familiar confounding, such as maternal chronic disease, this design could not account for familiar unshared factors that varied across pregnancies. Thus, residual confounding could still be a source of bias to consider. One potential confounder is maternal pre-pregnancy BMI [68]. However, we obtained similar results when additionally adjusting for pre-pregnancy BMI in women with available data (Table S16).

## Conclusion and implications

Our findings suggest that all types of maternal HDP, especially early-onset pre-eclampsia, are associated with increased risks of neurodevelopmental disorders (ADHD, ASD, and ID) in offspring. From a public health perspective, these findings highlight the importance of better management of maternal HDP and earlier screening and detection for neurodevelopmental disorders in their offspring.

## Supplementary Information

Below is the link to the electronic supplementary material.

Supplementary file1 (DOCX 233 kb)

## Data Availability

No additional data available.

## Abbreviations

HR: Hazard ratio
CI: Confidence interval
DNPR: Danish National Patient Register
DPCR: Danish Psychiatric Central Register
SNPR: Swedish National Patient Register
MBR: Medical Birth Registry
ICD-8: International Classification of Disease, Eighth Edition
ICD-10: International Classification of Disease, Tenth Edition
ADHD: Attention-deficit/hyperactivity disorder
ASD: Autism spectrum disorder
ID: Intellectual disability
HDP: Hypertensive disorders during pregnancy

## Code availability

The codes used in this study are available upon request.
