# Supplementary material for: Maternal hypertensive disorders and neurodevelopmental disorders in offspring: a population-based cohort in two Nordic countries
Source: Eur J Epidemiol. 2021 May 4;36(5):519–30. doi: 10.1007/s10654-021-00756-2 (PMC8159819; doi:10.1007/s10654-021-00756-2)
Supplement: Supplementary file 1 — Supplementary file1 (DOCX 233 kb) [file 10654_2021_756_MOESM1_ESM.docx]

Table S1 The description of registers used in the study

| Data source | Information | |
| --- | --- | --- |
| **Denmark** | |  |
| National Patient Register | | Information for all citizens on hospital discharge diagnoses since 1977, and outpatient diagnoses are included from 1995 |
| Medical Birth Register | | Information on gestational age, birth weight, Apgar score at 5 minutes, date of birth, sex, and singleton status since, and maternal smoking during pregnancy since 2004 |
| Danish Civil Registration System | | Information on individual personal identification number, place of birth, marital status, and vital statistics since 1981 |
| Integrated database for labour  market research | | Information on personal labour market affiliation and education establishments since 1981 |
| Psychiatric central research register | | Information of patients treated at psychiatric departments since 1970 |
| **Sweden** | |  |
| National Patient Register | | Information on diagnoses from all hospital admissions since 1987 and specialist outpatient care since 2001 |
| Medical Birth Register | | Information on complications during pregnancy and delivery, maternal age, height and weight, gestational age, birth weight, Apgar score at 5 minutes, date of birth, sex, singleton status, and maternal country of birth since 1973, and maternal smoking during pregnancy since 1982 |
| Education Register | | Information on highest level of completed formal education since 1985 |
| The Multi-Generation Register | | Information on biological relationships for all individuals residing in Sweden since 1961 |

Table S2 The diagnostic classification of hypertensive disorders during pregnancy in Denmark and Sweden

|  | In Denmark | In Sweden |
| --- | --- | --- |
| Chronic hypertension | *ICD-8* (1978-1993): 40009, 40019, 40029, 40039, 40099, 40199 and *ICD-10* (since 1994): I10, I11, I12, I13, I15, O10, O11 | *ICD-8* (1973-1986): 40199, 40299, 40399, 40499; *ICD-9* (1987-1996): 401, 402, 403, 404, 405, 642A-642C, 642H; and *ICD-10* (since 1997): I10, I11, I12, I13, I15, O11 |
| Gestational hypertension | *ICD-8*: 63700, 76029 and *ICD-10*: O13, O16 | *ICD-9*: 642D, 642X; and *ICD-10*: O13 |
| Pre-eclampsia* | *ICD-8*: 63703, 63704, 63709, 6371 and *ICD-10*: O14.0, O14.1, O14.2, O14.9, O15 | *ICD-9*: 642E-G, and *ICD-10*: O14, O15 |
| Moderate pre-eclampsia | *ICD-8*: 63703 and *ICD-10*: O14.0 | *ICD-9*: 642E and *ICD-10*: O14 (excluding O14.1) |
| Severe pre-eclampsia | *ICD-8*: 63704, 63709, 63719 and *ICD-10*: O14.1, O14.2, O14.9, O15 | *ICD-9*: 642F, 642G and *ICD-10*: O14.1, O15 |

ICD: International Statistical Classification of Diseases and Related Health Problems; *pre-eclampsia including eclampsia and the HELLP syndrome.

Table S3 The diagnostic classification of neurodevelopmental disorders in Denmark and Sweden

|  | In Denmark | In Sweden |
| --- | --- | --- |
| Attention-deficit/hyperactivity disorders | *ICD-8*: 308 and *ICD-10*: F90.0 and F98.8 | *ICD-9*: 314 and ICD-10: F90 |
| Autism spectrum disorders | *ICD-8*: 299 and *ICD-10*: F84 | *ICD-9*: 299 and ICD-10: F84 |
| Intellectual disability | *ICD-8*: 311, 312, 313, 314, 315 and *ICD-10*: F70-F79 | *ICD-9*: 317-319 and *ICD-10*: F70-F79 |

ICD: International Statistical Classification of Diseases and Related Health Problems.

Table S4 Associations between maternal hypertensive disorders during pregnancy and neurodevelopmental disorders in offspring stratified by sex

|  |  | | **Denmark** | |  | | **Sweden** | |
| --- | --- | --- | --- | --- | --- | --- | --- | --- |
| **Neurodevelopmental disorders** | | **No. of**  **Cases** | | **Adjusted^*^**  **HR (95% CI)** | | **No. of**  **Cases** | | **Adjusted^*^**  **HR (95% CI)** |
| **For ADHD** | |  | |  | |  | |  |
| **Boys** | |  | |  | |  | |  |
| No hypertension | | 22 997 | | 1.00 (ref) | | 41 242 | | 1.00 (ref) |
| Chronic hypertension | | 187 | | 1.01 (0.87-1.17) | | 243 | | 1.40 (1.23-1.59) |
| Gestational hypertension | | 193 | | 1.08 (0.93-1.24) | | 408 | | 1.12 (1.01-1.24) |
| Pre-eclampsia | | 821 | | 1.24 (1.15-1.33) | | 1502 | | 1.24 (1.18-1.31) |
| **Girls** | |  | |  | |  | |  |
| No hypertension | | 8632 | | 1.00 (ref) | | 17 010 | | 1.00 (ref) |
| Chronic hypertension | | 74 | | 1.12 (0.89-1.41) | | 80 | | 1.28 (1.02-1.59) |
| Gestational hypertension | | 66 | | 1.02 (0.80-1.30) | | 162 | | 1.20 (1.02-1.40) |
| Pre-eclampsia | | 288 | | 1.18 (1.05-1.34) | | 571 | | 1.21 (1.11-1.31) |
| **For ASD** | |  | |  | |  | |  |
| **Boys** | |  | |  | |  | |  |
| No hypertension | | 17 212 | | 1.00 (ref) | | 19 871 | | 1.00 (ref) |
| Chronic hypertension | | 178 | | 1.17 (1.01-1.35) | | 110 | | 1.10 (0.90-1.33) |
| Gestational hypertension | | 143 | | 0.96 (0.82-1.14) | | 226 | | 1.21 (1.06-1.39) |
| Pre-eclampsia | | 657 | | 1.27 (1.17-1.38) | | 806 | | 1.34 (1.25-1.44) |
| **Girls** | |  | |  | |  | |  |
| No hypertension | | 5324 | | 1.00 (ref) | | 7783 | | 1.00 (ref) |
| Chronic hypertension | | 58 | | 1.28 (0.99-1.66) | | 42 | | 1.20 (0.87-1.65) |
| Gestational hypertension | | 64 | | 1.43 (1.12-1.83) | | 75 | | 1.16 (0.92-1.46) |
| Pre-eclampsia | | 206 | | 1.34 (1.16-1.54) | | 312 | | 1.42 (1.27-1.60) |
| **For ID** | |  | |  | |  | |  |
| **Boys** | |  | |  | |  | |  |
| No hypertension | | 6561 | | 1.00 (ref) | | 10 052 | | 1.00 (ref) |
| Chronic hypertension | | 48 | | 1.23 (0.92-1.64) | | 73 | | 1.79 (1.41-2.27) |
| Gestational hypertension | | 74 | | 1.35 (1.06-1.73) | | 101 | | 1.09 (0.88-1.36) |
| Pre-eclampsia | | 279 | | 1.49 (1.32-1.69) | | 464 | | 1.73 (1.57-1.91) |
| **Girls** | |  | |  | |  | |  |
| No hypertension | | 3744 | | 1.00 (ref) | | 6312 | | 1.00 (ref) |
| Chronic hypertension | | 25 | | 1.08 (0.72-1.63) | | 32 | | 1.28 (0.89-1.85) |
| Gestational hypertension | | 37 | | 1.26 (0.90-1.75) | | 73 | | 1.42 (1.11-1.82) |
| Pre-eclampsia | | 157 | | 1.48 (1.25-1.75) | | 299 | | 1.84 (1.63-2.09) |

*Adjusted for birth year, parity, maternal age, maternal education, maternal cohabitation at birth, maternal origin, maternal history of psychiatric disorders before childbirth; ADHD: attention-deficit/hyperactivity disorder; ASD: autism spectrum disorder; ID: intellectual disability.

Table S5 Associations between maternal hypertensive disorders during pregnancy and neurodevelopmental disorders in offspring stratified by parity

|  |  | | **Denmark** | |  | | **Sweden** | |
| --- | --- | --- | --- | --- | --- | --- | --- | --- |
| **Neurodevelopmental disorders** | | **No. of**  **Cases** | | **Adjusted^*^**  **HR (95% CI)** | | **No. of**  **Cases** | | **Adjusted^*^**  **HR (95% CI)** |
| **For ADHD** | |  | |  | |  | |  |
| **Parity 1** | |  | |  | |  | |  |
| No hypertension | | 14 165 | | 1.00 (ref) | | 24 721 | | 1.00 (ref) |
| Chronic hypertension | | 95 | | 0.98 (0.80-1.20) | | 120 | | 1.41 (1.17-1.68) |
| Gestational hypertension | | 142 | | 1.01 (0.86-1.19) | | 323 | | 1.18 (1.06-1.33) |
| Pre-eclampsia | | 726 | | 1.19 (1.11-1.29) | | 1338 | | 1.18 (1.11-1.25) |
| **Parity 2 or more** | |  | |  | |  | |  |
| No hypertension | | 17 464 | | 1.00 (ref) | | 33 531 | | 1.00 (ref) |
| Chronic hypertension | | 166 | | 1.08 (0.92-1.26) | | 203 | | 1.34 (1.17-1.54) |
| Gestational hypertension | | 117 | | 1.13 (0.93-1.35) | | 247 | | 1.07 (0.94-1.22) |
| Pre-eclampsia | | 383 | | 1.28 (1.16-1.42) | | 735 | | 1.29 (1.20-1.39) |
| **For ASD** | |  | |  | |  | |  |
| **Parity 1** | |  | |  | |  | |  |
| No hypertension | | 11 256 | | 1.00 (ref) | | 12 901 | | 1.00 (ref) |
| Chronic hypertension | | 123 | | 1.39 (1.16-1.66) | | 65 | | 1.11 (0.86-1.44) |
| Gestational hypertension | | 130 | | 1.07 (0.90-1.27) | | 174 | | 1.14 (0.98-1.33) |
| Pre-eclampsia | | 564 | | 1.17 (1.07-1.28) | | 761 | | 1.31 (1.22-1.42) |
| **Parity 2 or more** | |  | |  | |  | |  |
| No hypertension | | 11 280 | | 1.00 (ref) | | 14 753 | | 1.00 (ref) |
| Chronic hypertension | | 113 | | 1.05 (0.87-1.26) | | 87 | | 1.14 (0.92-1.41) |
| Gestational hypertension | | 77 | | 1.06 (0.84-1.33) | | 127 | | 1.25 (1.04-1.49) |
| Pre-eclampsia | | 299 | | 1.56 (1.39-1.75) | | 357 | | 1.41 (1.27-1.58) |
| **For ID** | |  | |  | |  | |  |
| **Parity 1** | |  | |  | |  | |  |
| No hypertension | | 4218 | | 1.00 (ref) | | 6261 | | 1.00 (ref) |
| Chronic hypertension | | 30 | | 1.42 (0.99-2.05) | | 32 | | 1.50 (1.04-2.16) |
| Gestational hypertension | | 51 | | 1.19 (0.89-1.59) | | 85 | | 1.19 (0.94-1.50) |
| Pre-eclampsia | | 260 | | 1.44 (1.26-1.64) | | 471 | | 1.77 (1.61-1.96) |
| **Parity 2 or more** | |  | |  | |  | |  |
| No hypertension | | 6087 | | 1.00 (ref) | | 10 103 | | 1.00 (ref) |
| Chronic hypertension | | 43 | | 1.05 (0.77-1.43) | | 73 | | 1.65 (1.30-2.10) |
| Gestational hypertension | | 60 | | 1.43 (1.10-1.88) | | 89 | | 1.23 (0.98-1.54) |
| Pre-eclampsia | | 176 | | 1.54 (1.32-1.80) | | 292 | | 1.73 (1.53-1.96) |

*Adjusted for birth year, sex, maternal age, maternal education, maternal cohabitation at birth, maternal origin, maternal history of psychiatric disorders; ADHD: attention-deficit/hyperactivity disorder; ASD: autism spectrum disorder; ID: intellectual disability.

Table S6 Associations between maternal hypertensive disorders during pregnancy and neurodevelopmental disorders in offspring stratified by Apgar score at 5 minutes

|  |  | | **Denmark** | |  | | **Sweden** | |
| --- | --- | --- | --- | --- | --- | --- | --- | --- |
| **Neurodevelopmental disorders** | | **No. of**  **Cases** | | **Adjusted^*^**  **HR (95% CI)** | | **No. of**  **Cases** | | **Adjusted^*^**  **HR (95% CI)** |
| **For ADHD** | |  | |  | |  | |  |
| **Apgar score 10** | |  | |  | |  | |  |
| No hypertension | | 28 436 | | 1.00 (ref) | | 46 938 | | 1.00 (ref) |
| Chronic hypertension | | 218 | | 0.99 (0.87-1.13) | | 228 | | 1.34 (1.17-1.53) |
| Gestational hypertension | | 225 | | 1.05 (0.92-1.20) | | 444 | | 1.16 (1.05-1.28) |
| Pre-eclampsia | | 951 | | 1.24 (1.16-1.32) | | 1425 | | 1.21 (1.15-1.28) |
| **Apgar score <10** | |  | |  | |  | |  |
| No hypertension | | 2660 | | 1.00 (ref) | | 10 748 | | 1.00 (ref) |
| Chronic hypertension | | 39 | | 1.35 (0.98-1.85) | | 90 | | 1.36 (1.10-1.68) |
| Gestational hypertension | | 33 | | 1.14 (0.81-1.60) | | 122 | | 1.06 (0.88-1.28) |
| Pre-eclampsia | | 144 | | 1.05 (0.89-1.25) | | 612 | | 1.18 (1.09-1.29) |
| **For ASD** | |  | |  | |  | |  |
| **Apgar score 10** | |  | |  | |  | |  |
| No hypertension | | 20 129 | | 1.00 (ref) | | 22 146 | | 1.00 (ref) |
| Chronic hypertension | | 200 | | 1.17 (1.01-1.34) | | 95 | | 0.96 (0.77-1.18) |
| Gestational hypertension | | 185 | | 1.10 (0.95-1.27) | | 222 | | 1.15 (1.00-1.32) |
| Pre-eclampsia | | 738 | | 1.31 (1.21-1.41) | | 758 | | 1.34 (1.24-1.44) |
| **Apgar score <10** | |  | |  | |  | |  |
| No hypertension | | 1988 | | 1.00 (ref) | | 5230 | | 1.00 (ref) |
| Chronic hypertension | | 30 | | 1.22 (0.85-1.75) | | 57 | | 1.57 (1.20-2.05) |
| Gestational hypertension | | 21 | | 0.84 (0.54-1.30) | | 77 | | 1.35 (1.07-1.71) |
| Pre-eclampsia | | 112 | | 1.05 (0.86-1.27) | | 340 | | 1.34 (1.20-1.51) |
| **For ID** | |  | |  | |  | |  |
| **Apgar score 10** | |  | |  | |  | |  |
| No hypertension | | 8510 | | 1.00 (ref) | | 11 734 | | 1.00 (ref) |
| Chronic hypertension | | 57 | | 1.15 (0.88-1.50) | | 55 | | 1.31 (0.99-1.72) |
| Gestational hypertension | | 80 | | 1.19 (0.94-1.50) | | 109 | | 1.10 (0.90-1.36) |
| Pre-eclampsia | | 327 | | 1.45 (1.29-1.62) | | 433 | | 1.63 (1.47-1.80) |
| **Apgar score <10** | |  | |  | |  | |  |
| No hypertension | | 1532 | | 1.00 (ref) | | 4370 | | 1.00 (ref) |
| Chronic hypertension | | 16 | | 1.15 (0.69-1.92) | | 47 | | 1.81 (1.35-2.44) |
| Gestational hypertension | | 30 | | 1.54 (1.05-2.25) | | 65 | | 1.40 (1.07-1.82) |
| Pre-eclampsia | | 98 | | 1.17 (0.95-1.45) | | 304 | | 1.60 (1.41-1.81) |

*Adjusted for birthyear, sex, parity, maternal age, maternal education, maternal cohabitation at birth, maternal origin, maternal history of psychiatric disorders; ADHD: attention-deficit/hyperactivity disorder; ASD: autism spectrum disorder; ID: intellectual disability.

Table S7 Associations between maternal hypertensive disorders during pregnancy and neurodevelopmental disorders in offspring stratified by maternal age at the childbirth

|  |  | | **Denmark** | |  | | **Sweden** | |
| --- | --- | --- | --- | --- | --- | --- | --- | --- |
| **Neurodevelopmental disorders** | | **No. of**  **Cases** | | **Adjusted^*^**  **HR (95% CI)** | | **No. of**  **Cases** | | **Adjusted^*^**  **HR (95% CI)** |
| **For ADHD** | |  | |  | |  | |  |
| **Maternal age ≤30** | |  | |  | |  | |  |
| No hypertension | | 22 194 | | 1.00 (ref) | | 36 004 | | 1.00 (ref) |
| Chronic hypertension | | 128 | | 0.95 (0.80-1.13) | | 106 | | 1.18 (0.97-1.43) |
| Gestational hypertension | | 156 | | 0.98 (0.84-1.15) | | 311 | | 1.12 (1.00-1.26) |
| Pre-eclampsia | | 811 | | 1.21 (1.13-1.30) | | 1285 | | 1.17 (1.11-1.24) |
| **Maternal age >30** | |  | |  | |  | |  |
| No hypertension | | 9435 | | 1.00 (ref) | | 22 248 | | 1.00 (ref) |
| Chronic hypertension | | 133 | | 1.13 (0.95-1.34) | | 217 | | 1.43 (1.25-1.64) |
| Gestational hypertension | | 103 | | 1.19 (0.98-1.44) | | 259 | | 1.14 (1.00-1.29) |
| Pre-eclampsia | | 298 | | 1.25 (1.11-1.40) | | 788 | | 1.32 (1.23-1.42) |
| **For ASD** | |  | |  | |  | |  |
| **Maternal age ≤30** | |  | |  | |  | |  |
| No hypertension | | 13 818 | | 1.00 (ref) | | 15 129 | | 1.00 (ref) |
| Chronic hypertension | | 107 | | 1.26 (1.04-1.52) | | 40 | | 0.95 (0.69-1.32) |
| Gestational hypertension | | 108 | | 1.01 (0.84-1.23) | | 138 | | 1.14 (0.96-1.36) |
| Pre-eclampsia | | 529 | | 1.21 (1.11-1.32) | | 641 | | 1.38 (1.27-1.50) |
| **Maternal age >30** | |  | |  | |  | |  |
| No hypertension | | 8718 | | 1.00 (ref) | | 12 525 | | 1.00 (ref) |
| Chronic hypertension | | 129 | | 1.18 (0.99-1.41) | | 112 | | 1.22 (1.00-1.47) |
| Gestational hypertension | | 99 | | 1.15 (0.94-1.40) | | 163 | | 1.26 (1.08-1.48) |
| Pre-eclampsia | | 334 | | 1.43 (1.28-1.60) | | 477 | | 1.36 (1.24-1.49) |
| **For ID** | |  | |  | |  | |  |
| **Maternal age ≤30** | |  | |  | |  | |  |
| No hypertension | | 7177 | | 1.00 (ref) | | 9362 | | 1.00 (ref) |
| Chronic hypertension | | 32 | | 1.06 (0.74-1.52) | | 31 | | 1.55 (1.07-2.25) |
| Gestational hypertension | | 65 | | 1.29 (1.00-1.66) | | 87 | | 1.20 (0.94-1.51) |
| Pre-eclampsia | | 288 | | 1.36 (1.20-1.54) | | 441 | | 1.78 (1.60-1.97) |
| **Maternal age >30** | |  | |  | |  | |  |
| No hypertension | | 3128 | | 1.00 (ref) | | 7002 | | 1.00 (ref) |
| Chronic hypertension | | 41 | | 1.29 (0.94-1.76) | | 74 | | 1.62 (1.28-2.05) |
| Gestational hypertension | | 46 | | 1.35 (0.98-1.85) | | 87 | | 1.23 (0.98-1.55) |
| Pre-eclampsia | | 148 | | 1.81 (1.52-2.15) | | 322 | | 1.78 (1.58-2.01) |

*Adjusted for birth year, sex, parity, maternal education, maternal cohabitation at birth, maternal origin, maternal history of psychiatric disorders; ADHD: attention-deficit/hyperactivity disorder; ASD: autism spectrum disorder; ID: intellectual disability.

Table S8 Associations between maternal hypertensive disorders during pregnancy and neurodevelopmental disorders in offspring stratified by maternal psychiatric disorders at the childbirth

|  |  | | **Denmark** | |  | | **Sweden** | |
| --- | --- | --- | --- | --- | --- | --- | --- | --- |
| **Neurodevelopmental disorders** | | **No. of**  **Cases** | | **Adjusted^*^**  **HR (95% CI)** | | **No. of**  **Cases** | | **Adjusted^*^**  **HR (95% CI)** |
| **For ADHD** | |  | |  | |  | |  |
| **Maternal psychiatric disorder** | | | | | | | | |
| No hypertension | | 3525 | | 1.00 (ref) | | 4910 | | 1.00 (ref) |
| Chronic hypertension | | 36 | | 0.97 (0.69-1.35) | | 44 | | 1.30 (0.96-1.75) |
| Gestational hypertension | | 21 | | 0.89 (0.58-1.36) | | 39 | | 1.09 (0.79-1.50) |
| Pre-eclampsia | | 134 | | 1.38 (1.15-1.65) | | 165 | | 1.14 (0.97-1.34) |
| **No maternal psychiatric disorder** | | | | | | | | |
| No hypertension | | 28 104 | | 1.00 (ref) | | 53 342 | | 1.00 (ref) |
| Chronic hypertension | | 225 | | 1.02 (0.89-1.16) | | 279 | | 1.37 (1.22-1.55) |
| Gestational hypertension | | 238 | | 1.06 (0.93-1.20) | | 531 | | 1.14 (1.05-1.25) |
| Pre-eclampsia | | 975 | | 1.20 (1.12-1.28) | | 1908 | | 1.24 (1.18-1.30) |
| **For ASD** | |  | |  | |  | |  |
| **Maternal psychiatric disorder** | | | | | | | | |
| No hypertension | | 2230 | | 1.00 (ref) | | 2239 | | 1.00 (ref) |
| Chronic hypertension | | 28 | | 1.12 (0.77-1.63) | | 21 | | 1.05 (0.67-1.66) |
| Gestational hypertension | | 17 | | 0.99 (0.61-1.59) | | 28 | | 1.49 (1.01-2.19) |
| Pre-eclampsia | | 91 | | 1.43 (1.16-1.77) | | 80 | | 1.20 (0.95-1.51) |
| **No maternal psychiatric disorder** | | | | | | | | |
| No hypertension | | 20 306 | | 1.00 (ref) | | 25 415 | | 1.00 (ref) |
| Chronic hypertension | | 208 | | 1.22 (1.06-1.40) | | 131 | | 1.13 (0.95-1.35) |
| Gestational hypertension | | 190 | | 1.09 (0.94-1.26) | | 273 | | 1.17 (1.04-1.33) |
| Pre-eclampsia | | 772 | | 1.28 (1.19-1.38) | | 1038 | | 1.38 (1.29-1.47) |
| **For ID** | |  | |  | |  | |  |
| **Maternal psychiatric disorder** | | | | | | | | |
| No hypertension | | 911 | | 1.00 (ref) | | 1260 | | 1.00 (ref) |
| Chronic hypertension | | 7 | | 0.91 (0.43-1.92) | | 12 | | 1.36 (0.75-2.47) |
| Gestational hypertension | | 9 | | 1.14 (0.54-2.40) | | 14 | | 1.24 (0.68-2.25) |
| Pre-eclampsia | | 42 | | 1.66 (1.20-2.31) | | 51 | | 1.39 (1.02-1.89) |
| **No maternal psychiatric disorder** | | | | | | | | |
| No hypertension | | 9394 | | 1.00 (ref) | | 15 104 | | 1.00 (ref) |
| Chronic hypertension | | 66 | | 1.21 (0.94-1.55) | | 93 | | 1.65 (1.33-2.03) |
| Gestational hypertension | | 102 | | 1.33 (1.08-1.63) | | 160 | | 1.21 (1.02-1.44) |
| Pre-eclampsia | | 394 | | 1.47 (1.32-1.63) | | 712 | | 1.81 (1.67-1.96) |

*Adjusted for birth year, sex, parity, maternal education, maternal age, maternal cohabitation at birth, maternal origin; ADHD: attention-deficit/hyperactivity disorder; ASD: autism spectrum disorder; ID: intellectual disability.

Table S9 Associations between maternal hypertensive disorders during pregnancy and neurodevelopmental disorders in offspring excluding preterm birth, low birth weight or Apgar score 0-6

|  |  | | **Denmark** | |  | | **Sweden** | |
| --- | --- | --- | --- | --- | --- | --- | --- | --- |
| **Neurodevelopmental disorders** | | **No. of**  **Cases** | | **Adjusted^*^**  **HR (95% CI)** | | **No. of**  **Cases** | | **Adjusted^*^**  **HR (95% CI)** |
| **For ADHD** | |  | |  | |  | |  |
| No hypertension | | 28 685 | | 1.00 (ref) | | 52 958 | | 1.00 (ref) |
| Chronic hypertension | | 192 | | 0.98 (0.85-1.13) | | 215 | | 1.19 (1.04-1.36) |
| Gestational hypertension | | 224 | | 1.05 (0.92-1.20) | | 488 | | 1.12 (1.02-1.23) |
| Pre-eclampsia | | 805 | | 1.16 (1.08-1.24) | | 1360 | | 1.16 (1.10-1.22) |
| **For ASD** | |  | |  | |  | |  |
| No hypertension | | 20 661 | | 1.00 (ref) | | 24 933 | | 1.00 (ref) |
| Chronic hypertension | | 186 | | 1.19 (1.03-1.37) | | 94 | | 0.92 (0.75-1.13) |
| Gestational hypertension | | 184 | | 1.07 (0.93-1.24) | | 254 | | 1.17 (1.03-1.33) |
| Pre-eclampsia | | 659 | | 1.28 (1.18-1.39) | | 712 | | 1.26 (1.16-1.36) |
| **For ID** | |  | |  | |  | |  |
| No hypertension | | 8452 | | 1.00 (ref) | | 13 411 | | 1.00 (ref) |
| Chronic hypertension | | 49 | | 1.10 (0.82-1.46) | | 56 | | 1.26 (0.96-1.66) |
| Gestational hypertension | | 78 | | 1.18 (0.94-1.50) | | 116 | | 1.00 (0.81-1.22) |
| Pre-eclampsia | | 256 | | 1.24 (1.09-1.42) | | 369 | | 1.39 (1.25-1.56) |

*Adjusted for birth year, sex, parity, maternal age, maternal education, maternal cohabitation at birth, maternal origin, maternal history of psychiatric disorders before childbirth; ADHD: attention-deficit/hyperactivity disorder; ASD: autism spectrum disorder; ID: intellectual disability.

Table S10 Mediation analysis with gestational age as potential mediators between maternal hypertensive disorders during pregnancy and risk of neurodevelopmental disorders in offspring

| Neurodevelopmental disorders | Odds Ratio (95% CI)* |
| --- | --- |
| ADHD |  |
| Natural direct effect | 1.14 (1.10 to 1.17) |
| Natural indirect effect | 1.06 (1.05 to 1.06) |
| Total effect | 1.20 (1.17 to 1.23) |
| Proportion, %^b^ | 30.7 |
| ASD |  |
| Natural direct effect | 1.20 (1.15 to 1.25) |
| Natural indirect effect | 1.04 (1.04 to 1.05) |
| Total effect | 1.25 (1.21 to 1.30) |
| Proportion, % | 19.2 |
| ID |  |
| Natural direct effect | 1.30 (1.23 to 1.37) |
| Natural indirect effect | 1.15 (1.14 to 1.16) |
| Total effect | 1.50 (1.42 to 1.57) |
| Proportion, % | 34.9 |

*Adjusted birth year, sex, parity, maternal age, maternal education, maternal cohabitation at birth, maternal origin, maternal history of psychiatric disorders before childbirth; b: Proportion mediated was calculated as log (natural indirect effect)/log (total effect); ADHD: attention-deficit/hyperactivity disorder; ASD: autism spectrum disorder; ID: intellectual disability.

Table S11 Associations between maternal hypertensive disorders and neurodevelopmental disorders in offspring

| **Maternal hypertensive disorders** | **Combined** | |  | **Denmark** | |  | **Sweden** | |
| --- | --- | --- | --- | --- | --- | --- | --- | --- |
|  | Cases (No.) | aHR**^*^** (95% CI) |  | Cases (No.) | aHR* (95% CI) |  | Cases (No.) | aHR**^*^** (95% CI) |
| **ADHD** |  |  |  |  |  |  |  |  |
| No hypertensive disorders | 89 881 | 1.00 (ref) |  | 31 629 | 1.00 (ref) |  | 58 252 | 1.00 (ref) |
| Hypertensive disorders | 4595 | 1.24 (1.20-1.28) |  | 1629 | 1.16 (1.10-1.22) |  | 2966 | 1.23 (1.18-1.27) |
| Chronic hypertension | 429 | 1.14 (1.04-1.26) |  | 186 | 0.95 (0.83-1.10) |  | 243 | 1.28 (1.13-1.46) |
| Gestational hypertension | 829 | 1.13 (1.05-1.21) |  | 259 | 1.06 (0.94-1.20) |  | 570 | 1.14 (1.05-1.24) |
| Pre-eclampsia | 3182 | 1.27 (1.23-1.32) |  | 1109 | 1.22 (1.15-1.30) |  | 2073 | 1.23 (1.18-1.19) |
| Superimposed pre-eclampsia | 155 | 1.56 (1.33-1.82) |  | 75 | 1.33 (1.06-1.67) |  | 80 | 1.69 (1.36-2.11) |
| **ASD** |  |  |  |  |  |  |  |  |
| No hypertensive disorders | 50 190 | 1.00 (ref) |  | 22 536 | 1.00 (ref) |  | 27 654 | 1.00 (ref) |
| Hypertensive disorders | 2877 | 1.29 (1.24-1.34) |  | 1306 | 1.23 (1.16-1.30) |  | 1571 | 1.30 (1.24-1.37) |
| Chronic hypertension | 286 | 1.10 (0.98-1.24) |  | 172 | 1.12 (0.97-1.30) |  | 114 | 1.04 (0.86-1.26) |
| Gestational hypertension | 508 | 1.15 (1.05-1.26) |  | 207 | 1.07 (0.93-1.23) |  | 301 | 1.20 (1.06-1.35) |
| Pre-eclampsia | 1981 | 1.36 (1.30-1.42) |  | 863 | 1.29 (1.20-1.38) |  | 1118 | 1.36 (1.28-1.45) |
| Superimposed pre-eclampsia | 102 | 1.48 (1.21-1.80) |  | 64 | 1.44 (1.13-1.85) |  | 38 | 1.46 (1.05-2.01) |
| **ID** |  |  |  |  |  |  |  |  |
| No hypertensive disorders | 26 669 | 1.00 (ref) |  | 10 305 | 1.00 (ref) |  | 16 364 | 1.00 (ref) |
| Hypertensive disorders | 1662 | 1.58 (1.50-1.66) |  | 620 | 1.41 (1.30-1.54) |  | 1042 | 1.64 (1.53-1.75) |
| Chronic hypertension | 130 | 1.30 (1.09-1.55) |  | 49 | 1.02 (0.76-1.36) |  | 81 | 1.55 (1.23-1.94) |
| Gestational hypertension | 285 | 1.28 (1.13-1.45) |  | 111 | 1.32 (1.08-1.60) |  | 174 | 1.21 (1.03-1.43) |
| Pre-eclampsia | 1199 | 1.70 (1.60-1.81) |  | 436 | 1.49 (1.35-1.64) |  | 763 | 1.77 (1.64-1.92) |
| Superimposed pre-eclampsia | 48 | 1.85 (1.38-2.48) |  | 24 | 1.74 (1.15-2.62) |  | 24 | 1.85 (1.22-2.81) |

**^*^**Adjusted for sex, calendar year, parity, maternal age, maternal education, maternal cohabitation at birth, maternal history of psychiatric disorders before childbirth; aHR: adjusted hazard ratio; CI: confidence interval; No., number; ADHD: attention-deficit/hyperactivity disorder; ASD: autism spectrum disorder; ID: intellectual disability.

Table S12 Associations between maternal pre-eclampsia and neurodevelopmental disorders in offspring when using Swedish approach in defining pre-eclampsia (early-onset: before 34 completed gestational weeks)

|  | | **Neurodevelopmental disorders** | | | | | | | |
| --- | --- | --- | --- | --- | --- | --- | --- | --- | --- |
| **Maternal pre-eclampsia** | | **ADHD** | |  | **ASD** | |  | **ID** | |
|  | Sweden | | Denmark |  | Sweden | Denmark |  | Sweden | Denmark |
|  | aHR**^*^** (95% CI) | | aHR**^*^** (95% CI) |  | aHR**^*^** (95% CI) | aHR**^*^** (95% CI) |  | aHR**^*^** (95% CI) | aHR**^*^** (95% CI) |
| **Pre-eclampsia** | 1.23 (1.18-1.29) | | 1.22 (1.15-1.30) |  | 1.36 (1.28-1.45) | 1.29 (1.20-1.38) |  | 1.77 (1.64-1.92) | 1.49 (1.35-1.64) |
| **By timing of pre-eclampsia** | | | | | | | | | |
| Early-onset | 1.94 (1.71-2.19) | | 1.73 (1.45-2.06) |  | 2.13 (1.81-2.52) | 1.65 (1.18-2.30) |  | 4.54 (3.86-5.33) | 3.77 (3.00-4.74) |
| Late-onset | 1.16 (1.11-1.22) | | 1.18 (1.10-1.26) |  | 1.29 (1.21-1.38) | 1.27 (1.19-1.37) |  | 1.51 (1.39-1.65) | 1.31 (1.17-1.46) |

ADHD: attention-deficit/hyperactivity disorder; ASD: autism spectrum disorder; ID: intellectual disability; **^*^**Adjusted for sex, calendar year, parity, maternal age, maternal education, maternal cohabitation at birth, maternal history of psychiatric disorders before childbirth; aHR: adjusted hazard ratio; CI: confidence interval.

Table S13 Associations between maternal hypertensive disorders during pregnancy and neurodevelopmental disorders in offspring born after 1995 in Denmark and after 2001 in Sweden

|  |  | | **Denmark** | |  | | **Sweden** | |
| --- | --- | --- | --- | --- | --- | --- | --- | --- |
| **Neurodevelopmental disorders** | | **No. of**  **Cases** | | **Adjusted^*^**  **HR (95% CI)** | | **No. of**  **Cases** | | **Adjusted^*^**  **HR (95% CI)** |
| **For ADHD** | |  | |  | |  | |  |
| No hypertension | | 24 352 | | 1.00 (ref) | | 18 225 | | 1.00 (ref) |
| Chronic hypertension | | 239 | | 1.06 (0.93-1.21) | | 188 | | 1.36 (1.17-1.57) |
| Gestational hypertension | | 206 | | 1.15 (1.00-1.31) | | 190 | | 1.30 (1.13-1.50) |
| Pre-eclampsia | | 784 | | 1.20 (1.12-1.29) | | 725 | | 1.36 (1.26-1.47) |
| **For ASD** | |  | |  | |  | |  |
| No hypertension | | 17 457 | | 1.00 (ref) | | 9852 | | 1.00 (ref) |
| Chronic hypertension | | 224 | | 1.25 (1.10-1.43) | | 98 | | 1.16 (0.95-1.42) |
| Gestational hypertension | | 171 | | 1.17 (1.01-1.36) | | 115 | | 1.28 (1.06-1.54) |
| Pre-eclampsia | | 647 | | 1.31 (1.21-1.42) | | 418 | | 1.41 (1.27-1.55) |
| **For ID** | |  | |  | |  | |  |
| No hypertension | | 5196 | | 1.00 (ref) | | 4425 | | 1.00 (ref) |
| Chronic hypertension | | 58 | | 1.27 (0.98-1.65) | | 58 | | 1.48 (1.14-1.93) |
| Gestational hypertension | | 56 | | 1.54 (1.18-2.00) | | 44 | | 1.16 (0.86-1.58) |
| Pre-eclampsia | | 222 | | 1.73 (1.51-1.98) | | 244 | | 2.00 (1.75-2.28) |

*Adjusted for birth year, sex, parity, maternal age, maternal education, maternal cohabitation at birth, maternal origin, maternal history of psychiatric disorders before childbirth; ADHD: attention-deficit/hyperactivity disorder; ASD: autism spectrum disorder; ID: intellectual disability.

Table S14 Associations between maternal hypertensive disorders during pregnancy and neurodevelopmental disorders in offspring excluding diagnoses before the age of three years

|  |  | | **Denmark** | |  | | **Sweden** | |
| --- | --- | --- | --- | --- | --- | --- | --- | --- |
| **Neurodevelopmental disorders** | | **No. of**  **Cases** | | **Adjusted^*^**  **HR (95% CI)** | | **No. of**  **Cases** | | **Adjusted^*^**  **HR (95% CI)** |
| **For ADHD** | |  | |  | |  | |  |
| No hypertension | | 31 331 | | 1.00 (ref) | | 58 102 | | 1.00 (ref) |
| Chronic hypertension | | 257 | | 1.04 (0.92-1.17) | | 320 | | 1.36 (1.21-1.52) |
| Gestational hypertension | | 257 | | 1.07 (0.94-1.21) | | 568 | | 1.14 (1.05-1.24) |
| Pre-eclampsia | | 1090 | | 1.21 (1.14-1.29) | | 2067 | | 1.23 (1.17-1.29) |
| **For ASD** | |  | |  | |  | |  |
| No hypertension | | 21 754 | | 1.00 (ref) | | 26 952 | | 1.00 (ref) |
| Chronic hypertension | | 226 | | 1.19 (1.05-1.36) | | 142 | | 1.09 (0.92-1.29) |
| Gestational hypertension | | 201 | | 1.08 (0.94-1.24) | | 295 | | 1.20 (1.07-1.35) |
| Pre-eclampsia | | 829 | | 1.28 (1.19-1.37) | | 1095 | | 1.37 (1.28-1.45) |
| **For ID** | |  | |  | |  | |  |
| No hypertension | | 7865 | | 1.00 (ref) | | 14 796 | | 1.00 (ref) |
| Chronic hypertension | | 56 | | 1.14 (0.87-1.49) | | 95 | | 1.70 (1.38-2.10) |
| Gestational hypertension | | 87 | | 1.40 (1.12-1.74) | | 164 | | 1.30 (1.10-1.53) |
| Pre-eclampsia | | 335 | | 1.51 (1.35-1.70) | | 686 | | 1.77 (1.63-1.92) |

*Adjusted for birth year, sex, parity, maternal age, maternal education, maternal cohabitation at birth, maternal origin, maternal history of psychiatric disorders before childbirth; ADHD: attention-deficit/hyperactivity disorder; ASD: autism spectrum disorder; ID: intellectual disability.

Table S15 Associations between maternal hypertensive disorders during pregnancy and neurodevelopmental disorders in offspring using multiple imputation

|  |  | | **Denmark** | |  | | **Sweden** | |
| --- | --- | --- | --- | --- | --- | --- | --- | --- |
| **Neurodevelopmental disorders** | | **No. of**  **Cases** | | **Adjusted^*^**  **HR (95% CI)** | | **No. of**  **Cases** | | **Adjusted^*^**  **HR (95% CI)** |
| **For ADHD** | |  | |  | |  | |  |
| No hypertension | | 31 629 | | 1.00 (ref) | | 58 252 | | 1.00 (ref) |
| Chronic hypertension | | 261 | | 1.03 (0.91-1.16) | | 323 | | 1.34 (1.20-1.50) |
| Gestational hypertension | | 259 | | 1.04 (0.92-1.17) | | 570 | | 1.16 (1.07-1.26) |
| Pre-eclampsia | | 1109 | | 1.22 (1.15-1.30) | | 2073 | | 1.25 (1.19-1.30) |
| **For ASD** | |  | |  | |  | |  |
| No hypertension | | 22 536 | | 1.00 (ref) | | 27 654 | | 1.00 (ref) |
| Chronic hypertension | | 236 | | 1.18 (1.04-1.34) | | 152 | | 1.14 (0.97-1.34) |
| Gestational hypertension | | 207 | | 1.05 (0.91-1.20) | | 301 | | 1.19 (1.06-1.33) |
| Pre-eclampsia | | 863 | | 1.29 (1.21-1.39) | | 1118 | | 1.37 (1.29-1.45) |
| **For ID** | |  | |  | |  | |  |
| No hypertension | | 10 305 | | 1.00 (ref) | | 16 364 | | 1.00 (ref) |
| Chronic hypertension | | 73 | | 1.18 (0.93-1.48) | | 105 | | 1.57 (1.29-1.90) |
| Gestational hypertension | | 111 | | 1.35 (1.12-1.63) | | 174 | | 1.24 (1.06-1.44) |
| Pre-eclampsia | | 436 | | 1.48 (1.35-1.63) | | 763 | | 1.77 (1.64-1.90) |

*Adjusted for birth year, sex, parity, maternal age, maternal education, maternal cohabitation at birth, maternal origin, maternal history of psychiatric disorders before childbirth; ADHD: attention-deficit/hyperactivity disorder; ASD: autism spectrum disorder; ID: intellectual disability.

Table S16 Associations between maternal hypertensive disorders during pregnancy and neurodevelopmental disorders in offspring born after 2004 in Denmark and after 1992 in Sweden, including data on maternal body mass index (BMI)

|  |  | | **Denmark** | |  | | **Sweden** | |
| --- | --- | --- | --- | --- | --- | --- | --- | --- |
| **Neurodevelopmental disorders** | | **No. of**  **Cases** | | **Adjusted^*^**  **HR (95% CI)** | | **No. of**  **Cases** | | **Adjusted^*^**  **HR (95% CI)** |
| **For ADHD** | |  | |  | |  | |  |
| No hypertension | | 7226 | | 1.00 (ref) | | 58 089 | | 1.00 (ref) |
| Chronic hypertension | | 111 | | 1.13 (0.93-1.37) | | 317  56 | | 1.20 (1.06-1.36) |
| Gestational hypertension | | 63 | | 0.90 (0.70-1.16) | | 568 | | 1.04 (0.94-1.14) |
| Pre-eclampsia | | 245 | | 1.17 (1.02-1.33) | | 2059 | | 1.12 (1.06-1.18) |
| **For ASD** | |  | |  | |  | |  |
| No hypertension | | 5985 | | 1.00 (ref) | | 27 567 | | 1.00 (ref) |
| Chronic hypertension | | 109 | | 1.27 (1.04-1.55) | | 148 | | 0.99 (0.82-1.19) |
| Gestational hypertension | | 60 | | 0.95 (0.73-1.23) | | 300 | | 1.14 (1.00-1.31) |
| Pre-eclampsia | | 247 | | 1.40 (1.22-1.60) | | 1114 | | 1.26 (1.17-1.35) |
| **For ID** | |  | |  | |  | |  |
| No hypertension | | 1356 | | 1.00 (ref) | | 16 299 | | 1.00 (ref) |
| Chronic hypertension | | 19 | | 1.01 (0.63-1.61) | | 103 | | 1.52 (1.23-1.89) |
| Gestational hypertension | | 20 | | 1.60 (1.03-2.50) | | 174 | | 1.19 (0.99-1.43) |
| Pre-eclampsia | | 67 | | 1.86 (1.43-2.41) | | 760 | | 1.60 (1.46-1.76) |

*Adjusted for birthyear, sex, parity, maternal age, maternal education, maternal cohabitation at birth, maternal origin, maternal pregestational body mass index (BMI), maternal history of psychiatric disorders before childbirth; ADHD: attention-deficit/hyperactivity disorder; ASD: autism spectrum disorder; ID: intellectual disability.

Table S17 Associations between maternal hypertensive disorders during pregnancy (HDP) and neurodevelopmental disorders in offspring

|  | **Neurodevelopmental disorders** | | | | | | | |
| --- | --- | --- | --- | --- | --- | --- | --- | --- |
| **Maternal hypertensive disorders** | **ADHD** | |  | **ASD** | |  | **ID** | |
|  | Cases (No.) | aHR**^*^** (95% CI) |  | Cases (No.) | aHR**^*^** (95% CI) |  | Cases (No.) | aHR**^*^** (95% CI) |
| **HDP including chronic hypertension, gestational hypertension, and pre-eclampsia** | | | | | | | | |
| No HDP | 89 881 | 1.00 (ref) |  | 50 190 | 1.00 (ref) |  | 26 669 | 1.00 (ref) |
| HDP | 4595 | 1.24 (1.20-1.28) |  | 2877 | 1.29 (1.24-1.34) |  | 1662 | 1.58 (1.50-1.66) |
| **HDP including gestational hypertension and pre-eclampsia** | | | | | | | | |
| No HDP | 90 254 | 1.00 (ref) |  | 50 428 | 1.00 (ref) |  | 26 781 | 1.00 (ref) |
| HDP | 4222 | 1.26 (1.22-1.30) |  | 2639 | 1.32 (1.27-1.38) |  | 1550 | 1.61 (1.52-1.70) |

**^*^**Adjusted for sex, calendar year, parity, maternal age, maternal education, maternal cohabitation at birth, maternal history of psychiatric disorders before childbirth; aHR: adjusted hazard ratio; CI: confidence interval; No., number; ADHD: attention-deficit/hyperactivity disorders; ASD: autism spectrum disorders; ID: intellectual disability.

Table S18 Associations between maternal hypertensive disorders during pregnancy (HDP) and neurodevelopmental disorders in offspring

|  | **Neurodevelopmental disorders** | | | | | | | |
| --- | --- | --- | --- | --- | --- | --- | --- | --- |
| **Maternal hypertensive disorders** | **ADHD** | |  | **ASD** | |  | **ID** | |
|  | Cases (No.) | aHR**^*^** (95% CI) |  | Cases (No.) | aHR**^*^** (95% CI) |  | Cases (No.) | aHR**^*^** (95% CI) |
| **No HDP** | 89 881 | 1.00 (ref) |  | 50 190 | 1.00 (ref) |  | 26 669 | 1.00 (ref) |
| **HDP** | 4595 | 1.24 (1.20-1.28) |  | 2877 | 1.29 (1.24-1.34) |  | 1662 | 1.58 (1.50-1.66) |
| Chronic hypertension (Primary) | 200 | 1.08 (0.94-1.25) |  | 157 | 1.18 (1.00-1.38) |  | 69 | 1.43 (1.13-1.82) |
| Chronic hypertension (Secondary) | 384 | 1.33 (1.20-1.47) |  | 231 | 1.19 (1.04-1.36) |  | 109 | 1.40 (1.15-1.70) |
| Gestational hypertension | 829 | 1.14 (1.07-1.23) |  | 508 | 1.16 (1.06-1.27) |  | 285 | 1.29 (1.14-1.46) |
| Pre-eclampsia | 3182 | 1.28 (1.24-1.33) |  | 1981 | 1.37 (1.30-1.43) |  | 1199 | 1.71 (1.61-1.81) |

**^*^**Adjusted for sex, calendar year, parity, maternal age, maternal education, maternal cohabitation at birth, maternal history of psychiatric disorders before childbirth; aHR: adjusted hazard ratio; CI: confidence interval; No., number; ADHD: attention-deficit/hyperactivity disorders; ASD: autism spectrum disorders; ID: intellectual disability.

Table S19 Associations between maternal hypertensive disorders during pregnancy (HDP) and neurodevelopmental disorders in offspring

|  | **Neurodevelopmental disorders** | | | | | | | |
| --- | --- | --- | --- | --- | --- | --- | --- | --- |
| **Maternal hypertensive disorders** | **ADHD** | |  | **ASD** | |  | **ID** | |
|  | Cases (No.) | aHR**^*^** (95% CI) |  | Cases (No.) | aHR**^*^** (95% CI) |  | Cases (No.) | aHR**^*^** (95% CI) |
| **No HDP** | 89 881 | 1.00 (ref) |  | 50 190 | 1.00 (ref) |  | 26 669 | 1.00 (ref) |
| **HDP** | 4595 | 1.24 (1.20-1.28) |  | 2877 | 1.29 (1.24-1.34) |  | 1662 | 1.58 (1.50-1.66) |
| Chronic hypertension (Primary) | 200 | 1.05 (0.91-1.21) |  | 157 | 1.15 (0.98-1.34) |  | 69 | 1.41 (1.11-1.79) |
| Chronic hypertension (Secondary) | 384 | 1.29 (1.17-1.43) |  | 231 | 1.16 (1.02-1.32) |  | 109 | 1.37 (1.13-1.67) |
| Gestational hypertension | 829 | 1.14 (1.06-1.22) |  | 508 | 1.16 (1.06-1.27) |  | 285 | 1.29 (1.14-1.46) |
| Pre-eclampsia | 3182 | 1.27 (1.23-1.32) |  | 1981 | 1.36 (1.30-1.42) |  | 1199 | 1.70 (1.60-1.81) |

**^*^**Adjusted for sex, calendar year, parity, maternal age, maternal education, maternal cohabitation at birth, maternal history of psychiatric disorders before childbirth, and Charlson comorbidity index (CCI); aHR: adjusted hazard ratio; CI: confidence interval; No., number; ADHD: attention-deficit/hyperactivity disorders; ASD: autism spectrum disorders; ID: intellectual disability.

Figure S1 Cumulative risk for neurodevelopmental disorders in children after exposure to maternal hypotensive disorders during pregnancy in Denmark

Figure S2 Cumulative risk for neurodevelopmental disorders in children after exposure to maternal hypotensive disorders during pregnancy in Sweden
